# Clinical significance of cytokeratin 19 fragment in COVID-19 patients: a retrospective study

**DOI:** 10.3389/fpubh.2025.1738947

**Published:** 2025-12-18

**Authors:** Simei Shen, Dandan Wu, Haiqin Xie, Haiyan He, Yihua Wang, Xuedong Lv

**Affiliations:** 1 Department of Pulmonary and Critical Care Medicine, Nantong First People’s Hospital, Nantong, Jiangsu, China; 2 Biological Sciences, Faculty of Environmental and Life Sciences, University of Southampton, Southampton, United Kingdom; 3 Institute for Life Sciences, University of Southampton, Southampton, United Kingdom; 4 NIHR Southampton Biomedical Research Centre, University Hospital Southampton, Southampton, United Kingdom

**Keywords:** cancer biomarkers, COVID-19, CYFRA 21-1, risk stratification, viral pneumonia

## Abstract

**Background:**

Cytokeratin 19 fragment (CYFRA 21-1) is an important biomarker of lung cancer. There are clinical observations of elevated serum levels of lung cancer biomarkers in patients with viral pneumonia. However, the clinical significance of CYFRA 21-1 in coronavirus disease 2019 pneumonia has not been investigated.

**Methods:**

This retrospective study included 252 patients with community-acquired pneumonia (CAP) between December 1, 2022, and September 30, 2023. They were classified into three groups by clinical diagnosis and severity, namely mild non-COVID-19 CAP (*n* = 86), mild COVID-19 (*n* = 100), and severe COVID-19 (*n* = 66). Demographic characteristics, history, outcomes, and laboratory tests, including CYFRA 21-1 levels, were collected and compared among the groups. Risk factors associated with the diagnosis of COVID-19 pneumonia and severity were explored using appropriate statistical methods.

**Results:**

CYFRA 21-1 levels progressively increased from mild non–COVID-19 CAP to mild COVID-19 and severe COVID-19. Lower lymphocyte and platelet counts, alongside elevated CYFRA 21-1 levels, were associated with COVID-19 pneumonia. Multivariate analysis identified CYFRA 21-1 as an independent diagnostic [diagnosis odds ratio (OR) = 2.369; 95% confidence interval (CI) = 1.638–3.605; *p* < 0.001] and prognosis factor of COVID-19 pneumonia (severity OR = 1.416; 95% CI = 1.119–1.867; *p* = 0.01). The area under the receiver operating characteristic curve of CYFRA 21-1 for predicting the development of severe COVID-19 pneumonia was 0.913. Spearman analysis showed a negative correlation between CYFRA 21-1 levels and oxygenation index, with a correlation coefficient of −0.278 (*p* = 0.024).

**Conclusion:**

CYFRA 21-1 may be a potential diagnostic and prognostic indicator of COVID-19 pneumonia. Prospective multicenter studies are needed to confirm its clinical value.

## Introduction

The coronavirus disease 2019 (COVID-19) is a contagious disease caused by severe acute respiratory syndrome coronavirus 2 (SARS-CoV-2) infection. The initial case of COVID-19 was identified in Wuhan, China, in 2019, and the disease rapidly spread worldwide. According to the World Health Organization (WHO), by December 2023, approximately 7 million deaths and over 700 million confirmed cases of COVID-19 had been reported worldwide (1). Given its highly variable nature, SARS-CoV-2 has not been eliminated. Sporadic infections and occasionally localized outbreaks still pose a great threat to human health (2).

Although COVID-19 can affect multiple systems, the respiratory system is the most commonly affected. Clinical manifestations vary widely, ranging from mild flu-like illness, and moderate pneumonia to severe, even life-threatening conditions. Diagnosis predominantly relies on the detection of SARS-CoV-2 virus, with real-time reverse-transcription PCR (RT-PCR) remaining the most common method (3). However, many COVID-19 patients often test negative for SARS-CoV-2, whether due to false negatives or genuinely low viral loads, which complicates timely and accurate diagnosis. As with other viral pneumonias, such as those caused by influenza and respiratory syncytial virus, severe COVID-19 pneumonia is primarily due to an excessive immune response rather than direct damage caused by the virus itself (4–6). Additionally, while some individuals test positive for SARS-CoV-2, their pneumonia might be attributable to co-infections with other pathogens (7). These factors can sometimes make it challenging to distinguish COVID-19 from typical community-acquired pneumonia (CAP), especially among older adults and children, who are also vulnerable populations to COVID-19. Timely diagnosis is crucial not only for preventing the progression to severe stages but also for avoiding the misuse of antibiotics (8).

The well-established risk factors for the development of severe COVID-19 include advanced age, preexisting comorbidities, and a compromised immune system (9–11). However, the clinical outcomes within these populations may still show considerable variations. Moreover, cases of severe COVID-19 can even occur in generally healthy populations. Thus, accurate risk stratification is challenging, and effective strategies are urgently needed to predict and prevent severe COVID-19. It has been reported that serum levels of cancer biomarkers are elevated in patients with severe COVID-19 compared with mild COVID-19 cases (12, 13). Cytokeratin 19 fragment (CYFRA 21-1), a common lung cancer–related biomarker, is elevated in bronchoalveolar lavage fluid of patients with acute respiratory distress syndrome (ARDS), as well as in the serum of patients with interstitial lung diseases and radiation pneumonitis (14–17). CYFRA 21-1 is released after the proteolytic degradation of cytokeratin 19. Aberrant accumulation of CYFRA 21-1 represents apoptosis or necrosis of a wide range of epithelial cells (18). However, it remains unknown whether CYFRA 21-1 level is related to COVID-19.

Therefore, in this study, we collected clinical data from patients diagnosed with typical CAP and those with COVID-19 penumonia. We aimed to evaluate the diagnostic and prognostic value of CYFRA 21-1 in COVID-19 patients.

## Materials and methods

### Participants

The study was conducted in accordance with the Declaration of Helsinki and the International Ethical Guidelines for Biomedical Research involving Human Subjects (CIMOS). This study involved a retrospective analysis performed at the Department of Pulmonary and Critical Care Medicine in Nantong First People’s Hospital, which was approved by the Ethics Committee of the Nantong First People’s Hospital, Jiangsu, China (Approval No.: 2024KT105). All hospitalized adult patients (age: ≥18 years) diagnosed with CAP from December 1, 2022, to September 30, 2023, were enrolled. Patients with a history of structural pulmonary diseases or tumors were excluded, as these conditions could affect the levels of lung cancer biomarkers (detailed in Supplementary Figure S1). At least three senior respiratory physicians assessed the diagnosis and disease severity in line with relevant guidelines (19, 20). The diagnosis of COVID-19 pneumonia was based on a comprehensive evaluation of exposure history, clinical symptoms, laboratory tests, chest imaging findings, and response to treatment. Although the WHO classifies any COVID-19 patient with radiographic pneumonia as having at least moderate disease, in the present study, we stratified the patients according to the clinical severity of pneumonia, based on the severity criteria recommended in the CAP guidelines (19). This approach was adopted to ensure that COVID-19 pneumonia and bacterial CAP could be compared at similar levels of physiological severity. Patients who did not meet the criteria for severe pneumonia were assigned to the mild COVID-19 pneumonia group, while those meeting one major criterion or at least three minor criteria were classified into the severe COVID-19 group. Minor criteria were as follows: (1) respiratory rate ≥ 30 breaths/min; (2) oxygenation index (OI, defined as the PaO2/FiO2 ratio) ≤ 250; (3) confusion or disorientation; (4) blood urea nitrogen level ≥ 20 mg/dL; (5) chest imaging showing multilobar infiltration or progression >50% within 24 to 48 h; and (6) hypotension requiring aggressive fluid resuscitation. Major criteria were as follows: (1) shock requiring vasopressors and (2) respiratory failure requiring mechanical ventilation.

### Measures

Patient demographic characteristics, body mass index (BMI), pre-hospital history, laboratory tests, and clinical outcomes were collected from archived medical records from May 4 to May 10, 2024. After data collection, none of the authors had access to information that could identify individual participants.

PaO_2_ levels were obtained from arterial blood gas analysis conducted on the day of the patient’s admission. Lymphocyte and platelet counts were measured from venous blood samples also taken on the day of admission. The levels of the following biomarkers were determined from venous blood samples collected the following morning after a 12-h fasting period post-admission: progastrin-releasing peptide (ProGRP), cytokeratin 19 fragment (CYFRA 21-1), neuron-specific enolase (NSE), carcinoembryonic antigen (CEA), squamous cell carcinoma antigen (SCCA), C-reactive protein (CRP), alanine aminotransferase (ALT), aspartate aminotransferase (AST), blld urea nitrogen (BUN), creatinine (Cr), B-type natriuretic peptide precursor (proBNP) and erythrocyte sedimentation rate (ESR).

### Statistical analysis

Normality of continuous variables was assessed using the Shapiro–Wilk test. As most variables did not comply with normal distributions, continuous data were summarized as median [interquartile range (IQR)] and were analyzed with the Mann–Whitney *U* test to assess the difference between the two groups. Significant differences among the three groups were estimated using the Kruskal–Wallis test followed by Dunn’s *post hoc* analysis. Categorical variables were expressed as *n* (%) and were compared using Fisher’s exact test. Variables that showed significant differences between groups were considered for subsequent univariate logistic regression analyses. Candidate variables with *p* < 0.05 in the univariate analyses were entered into the multivariate logistic regression models. Multicollinearity was assessed using the variance inflation factors (VIF), ensuring that all of the included predictors included had a VIF lower than 3. Spearman rank correlation was used to evaluate the association between CYFRA 21-1 and the OI (PaO₂/FiO₂). *p* values less than 0.05 were considered statistically significant. All data analyses and graph creation were performed using GraphPad Prism (version 10.1.1) and IBM SPSS Statistics (version 29.0).

## Results

### Patients’ characteristics

A total of 86 adult patients diagnosed with regular CAP and 166 patients with COVID-19 pneumonia between December 1, 2022, and September 30, 2023, were enrolled in this study. Among the COVID-19 cases, 100 were classified as mild and 66 as severe COVID-19. There were no significant differences observed in BMI, smoking history, hypertension, diabetes, or coronary heart disease among the groups. Age and sex distributions were comparable between the mild COVID-19 and non–COVID-19 groups, whereas the severe COVID-19 group comprised significantly older individuals and a lower proportion of females.

Microbiological identification was limited in the CAP cohort, given that routine sputum cultures were negative in most of these patients. Nevertheless, their overall clinical presentation, including neutrophil-predominant leukocytosis, elevated procalcitonin levels, characteristic radiologic findings, and, most importantly, clear and timely improvement following empirical antibacterial therapy, was highly consistent with bacterial pneumonia. Confirmed pathogens were available for a small subset of patients and included *Mycoplasma pneumoniae*, *Streptococcus pneumoniae,* and *Haemophilus influenzae.*

Lymphocyte and platelet counts were generally lower in COVID-19 patients, regardless of disease severity. Surprisingly, platelet counts did not significantly differ between the mild and severe COVID-19 groups. The mild COVID-19 group and the regular CAP group exhibited no significant changes in inflammatory markers, including CRP and ESR. Furthermore, there were no substantial changes in liver function indicators (ALT and AST), renal function indicators (BUN and Cr), or heart failure marker proBNP. Importantly, OI (defined as the PaO_2_/FiO_2_ ratio) values were comparable between patients in the mild CAP and mild COVID-19 groups. Taken together, these findings suggested that the disease severity was similar in both groups. In contrast, the patients with severe COVID-19 exhibited significantly elevated levels of inflammatory biomarkers, along with marked declines in cardiac, hepatic, and renal functions, and OI (all *p* < 0.05).

There were remarkable differences in CYFRA 21-1 and CEA levels between the mild COVID-19 group and the CAP group, while other biomarkers showed no significant differences. Notably, all of the examined lung cancer–related biomarkers significantly differed between the mild and severe COVID-19 groups. These findings are summarized in Table 1.

**Table 1:** Patients’ characteristics.

Variable	Mild non−COVID-19 CAP (*n* = 86)	Mild COVID-19 pneumonia (*n* = 100)	Severe COVID-19 pneumon*i*a (n = 66)	*p-*value Mild non−COVID-19 CAP vs. Mild COVID-19	*p-*value Mild COVID-19 vs. Severe COVID-19
Age					
	65.50 (57.7–73.00)	67.50 (57.25–79.00)	78.00 (73.00–83.00)	0.11	1.1 × 10^−5^*
Sex					
Female	41 (47.67%)	54 (54.00%)	13 (19.70%)	0.46	< 1 × 10^−4^*
Male	45 (52.33%)	46 (46.00%)	53 (80.30%)		
BMI					
	24.22 (21.06–26.13)	23.73 (21.61–26.02)	23.53 (21.78–25.99)	0.89	0.67
Disease history					
Hypertension	42 (48.84%)	50 (50.00%)	39 (59.09%)	0.88	0.27
Diabetes	17 (19.77%)	21 (21.00%)	20 (30.30%)	0.86	0.20
CHD	2 (2.33%)	5 (5.00%)	8 (12.12%)	0.45	0.14
Smoke history					
	7 (8.14%)	5 (5.00%)	6 (9.09%)	0.55	0.35
Tumor biomarkers					
ProGRP (pg/mL)	34.00 (24.00–43.50)	37.50 (26.25–49.00)	65.00 (41.00–96.00)	0.16	6.7 × 10^−8^*
CYFRA 21-1 (ng/mL)	1.50 (1.09–1.99)	2.53 (1.67–3.63)	11.37 (5.44–16.18)	8.2 × 10^−10^*	2.3 × 10^−19^*
NSE (ng/mL)	12.05 (9.64–14.53)	12.80 (10.53–16.40)	25.20 (18.15–32.03)	0.10	3.3 × 10^−16^*
CEA (ng/mL)	1.50 (0.87–2.28)	2.17 (1.42–3.72)	5.93 (3.60–11.38)	1.7 × 10^−5^*	4.3 × 10^−12^*
SCCA (ng/mL)	1.17 (0.85–1.85)	1.02 (0.68–1.89)	1.32 (0.82–2.90)	0.11	1.1 × 10^−9^*
Laboratory test					
Lymphocyte ( × 10^9^/L)	1.40 (1.18–1.90)	1.05 (0.80–1.38)	0.50 (0.38–0.70)	1.2 × 10^−5^*	8.9 × 10^−14^*
Platelets ( × 10^9^/L)	217.50 (183.75–254.50)	180.00 (124.25–226.00)	182.50 (122.50–222.25)	1.7 × 10^−4^*	0.78
CRP (mg/L)	27.58 (9.56–56.14)	28.23 (9.56–56.14)	127.67 (73.99–177.32)	0.94	1.3 × 10^−14^*
ESR (mm)	29.50 (16.00–42.00)	29.50 (19.25–55.00)	49.00 (36.00–66.75)	0.27	4.0 × 10^−5^*
ALT (U/L)	19.00 (15.00–25.00)	19.00 (14.00–32.75)	31.00 (19.95–48.00)	0.73	2.9 × 10^−4^*
AST (U/L)	18.00 (15.00–25.00)	23.00 (16.00–33.00)	35.95 (26.25–62.50)	0.81	2.1 × 10^−11^*
proBNP (pg/mL)	85.45 (31.48–248.28)	125.45 (61.05–311.45)	763.75 (396.13–1949.75)	0.09	7.9 × 10^−15^*
BUN (mmol/L)	4.47 (3.54–5.72)	4.68 (3.65–5.70)	7.40 (5.64–10.02)	0.29	3.9 × 10^−9^*
Cr (μmol/L)	58.45 (53.08–74.78)	66.65 (55.13–78.13)	82.10 (50.00–117.78)	0.06	0.08
OI	380.48 (340.80–416.67)	366.67 (334.46–409.88)	187.29 (130.04–232.78)	0.30	4.8 × 10^−27^*
Outcome					
Death	0	0	34 (51.2%)		

CHD, chronic heart diseases; ProGRP, progastrin-releasing peptide (ProGRP); CYFRA 21-1, cytokeratin 19 fragment; NSE, neuron-specific enolase (NSE); CEA, carcinoembryonic antigen; SCCA, squamous cell carcinoma antigen; CRP, C-reactive protein; ESR, erythrocyte sedimentation rate; ALT, alanine aminotransferase; AST, aspartate aminotransferase; proBNP, B-type natriuretic peptide precursor; BUN, blood urea nitrogen; Cr, creatinine; and OI, Oxygenation index. Death refers to in-hospital mortality.

Data are shown as median (IQR) or *n* (%). *p-value < 0.05 with statistical significance.

### CYFRA 21-1 discriminates between regular CAP and COVID-19 pneumonia

**Figure 1:**
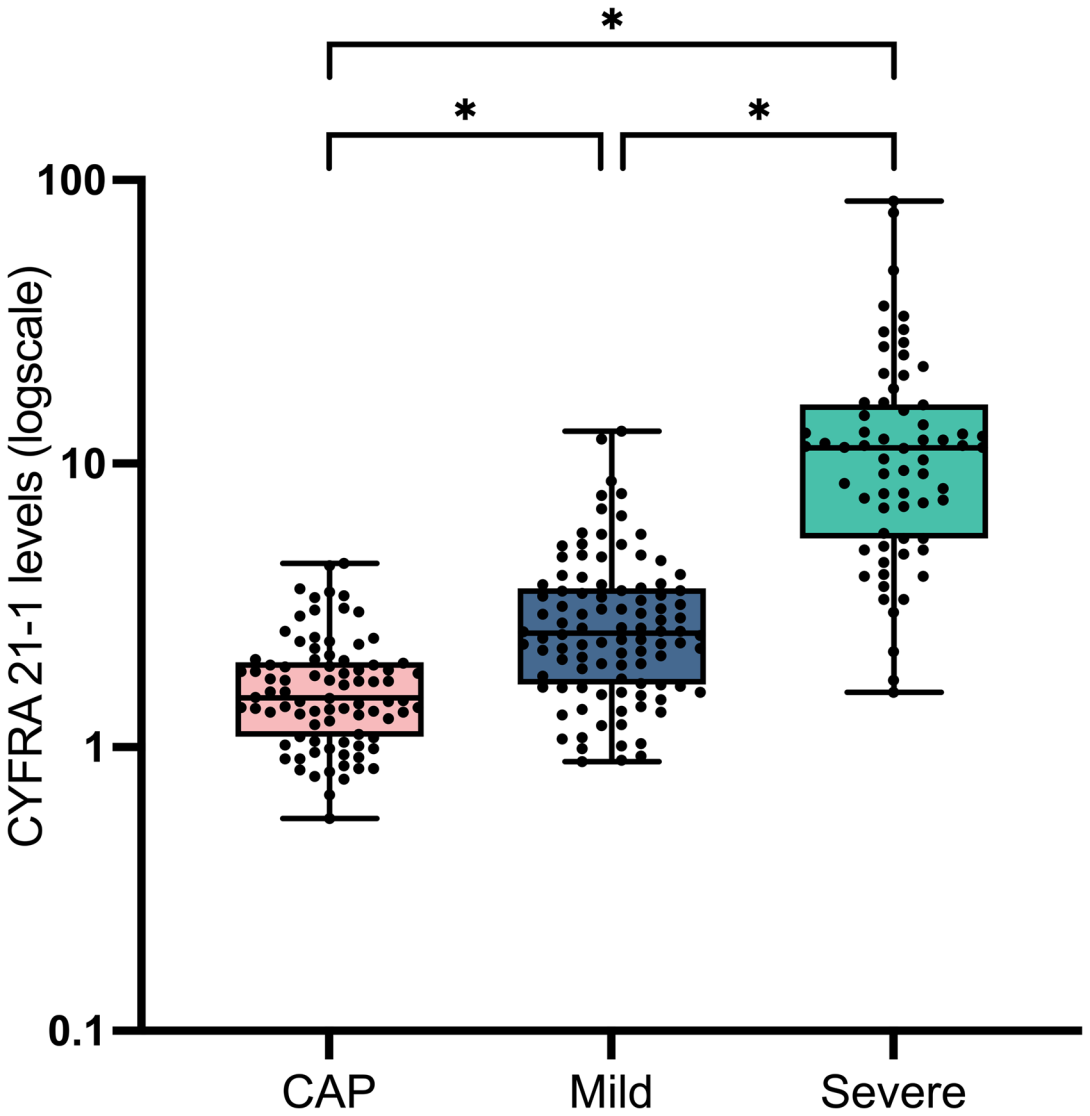
CYFRA 21-1 levels in non–COVID-19 community-acquired pneumonia (CAP) and mild or severe COVID-19 pneumonia. Data are shown as median and range, with individual values indicated. *p-value < 0.05 with statistical significance.

To explore the diagnostic value of lung cancer-related biomarkers in COVID-19 pneumonia, a multivariate logistic regression analysis was conducted. As shown in Table 2, CYFRA 21-1 was identified as an independent risk factor of COVID-19 pneumonia [odds ratio (OR) = 2.369; 95% confidence interval (CI) = 1.638–3.605; *p* < 0.001]. Figure 1 illustrates a progressive increase in CYFRA 21-1 levels from non-COVID-19 CAP and mild COVID-19 to severe COVID-19, with statistically significant differences observed among the groups (all *p* < 0.001). This trend highlighted the diagnostic potential of CYFRA 21-1 in distinguishing COVID-19 pneumonia from regular CAP. Moreover, there was a dramatic rise in CYFRA 21-1 levels in the severe COVID-19 group, suggesting a possible correlation with COVID-19 severity.

**Table 2:** Multivariate logistic regression analysis for risk factors associated with COVID-19 pneumonia.

Variable	OR (odds ratio)	95% CI (confidence interval)	*p* value
Tumor biomarkers			
CYFRA 21-1	2.369	1.638–3.605	< 0.001*
CEA	1.110	0.952–1.331	0.21
Laboratory test			
Lymphocyte	0.569	0.305–1.030	0.07
Platelets	0.9942	0.986–0.997	0.003*

CYFRA 21-1, cytokeratin 19 fragment; and CEA, carcinoembryonic antigen.

*p-value < 0.05 with statistical significance.

### CYFRA 21-1 is associated with the severity of COVID-19 pneumonia

**Figure 2:**
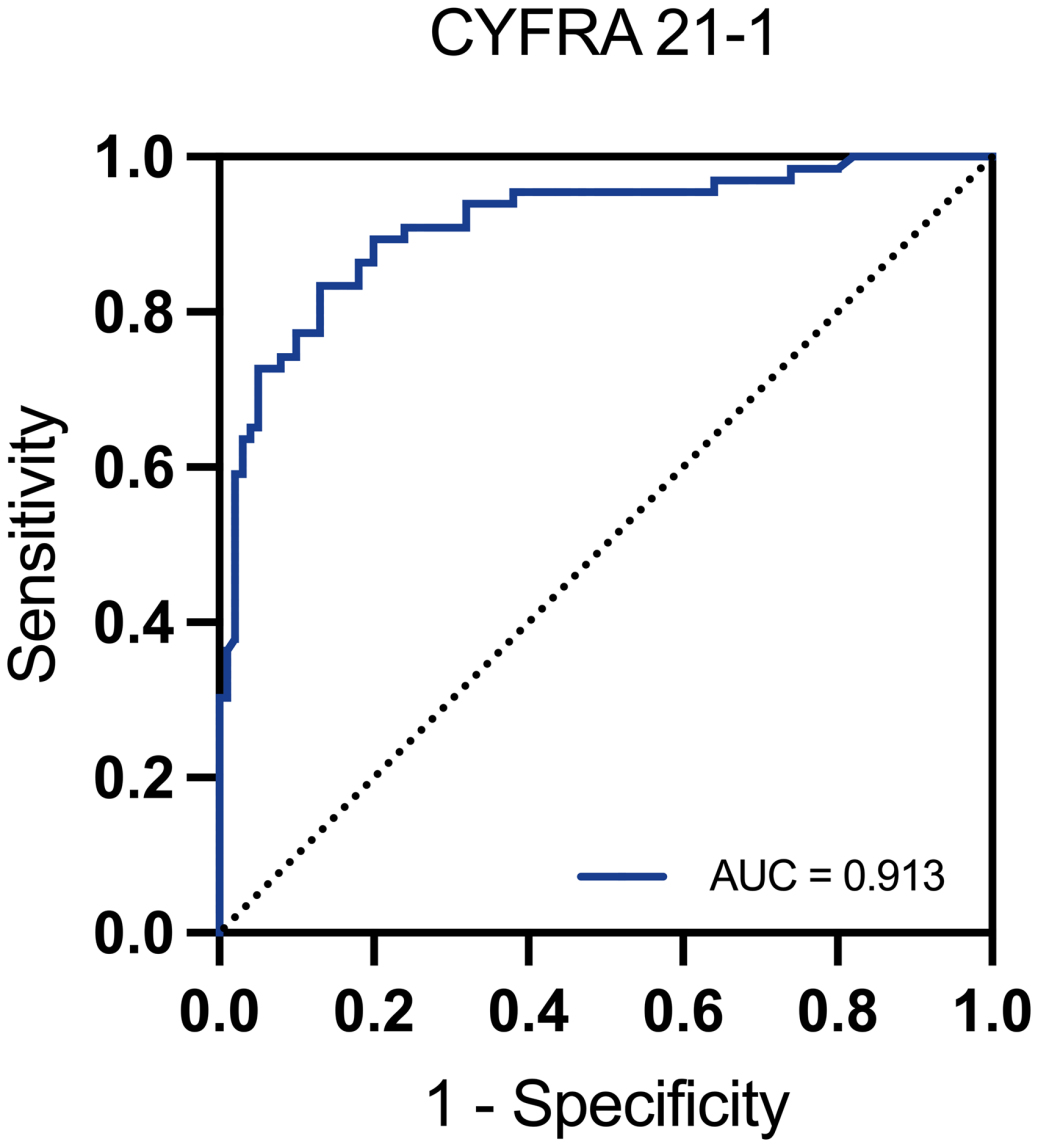
Receiver operating characteristic (ROC) curve for CYFRA 21-1 to predict the development of severe COVID-19 pneumonia. AUC, the area under the curve.

To further ascertain whether CYFRA 21-1 levels correlate with the severity of COVID-19 pneumonia, univariate and multivariate logistic regression analyses were conducted within the mild and severe COVID-19 patient cohorts. As shown in Table 3, the risk factors associated with severe COVID-19 pneumonia included male sex, elevated levels of CYFRA 21-1 and CEA, decreased lymphocyte counts, and increased CRP levels. CYFRA 21-1 was an independent risk factor of severe COVID-19 pneumonia (OR = 1.416; 95% CI = 1.119–1.867; *p* = 0.01). In addition, the area under the receiver operating characteristic curve of CYFRA 21-1 for predicting the development of severe COVID-19 pneumonia was 0.913, with a 95% CI of 0.867–0.960 (*p* < 0.001, Figure 2). Taken together, these findings indicate that CYFRA 21-1 could be used as an effective predictor of severe COVID-19 pneumonia.

**Table 3:** Multivariate logistic regression analysis for risk factors associated with the severity of COVID-19 pneumonia.

Variable	OR (odds ratio)	95% CI (confidence interval)	*p* value
Sex			
	5.848	1.256–35.680	0.04*
Age			
	1.065	0.994–1.155	0.10
Tumor biomarkers			
ProGRP	1.013	0.992–1.037	0.23
CYFRA 21-1	1.416	1.119–1.867	0.01*
NSE	1.069	1.008–1.143	0.03*
CEA	0.982	0.855–1.117	0.78
SCCA	1.029	0.850–1.261	0.79
Laboratory test			
Lymphocyte	0.046	0.004–0.364	0.01*
CRP	1.015	1.003–1.029	0.03*
ESR	1.007	0.976–1.038	0.66
proBNP	1.000	1.000–1.001	0.30
BUN	0.906	0.725–1.166	0.41

ProGRP, progastrin-releasing peptide (ProGRP); CYFRA 21-1, cytokeratin 19 fragment; NSE, neuron-specific enolase (NSE); CEA, carcinoembryonic antigen; SCCA, squamous cell carcinoma antigen; CRP, C-reactive protein; ESR, erythrocyte sedimentation rate; proBNP, B-type natriuretic peptide precursor; and BUN, blood urea nitrogen.

^*^p-value < 0.05 with statistical significance.

### CYFRA 21-1 is an independent predictor of mortality in severe COVID-19 pneumonia

Comparisons of demographic and clinical characteristics between survivors and non-survivors were conducted within the severe COVID-19 cohort. As indicated in Table 4, the non-survivors group exhibited a lower proportion of females and higher levels of CYFRA 21-1. As expected, lower levels of AST, BUN, and creatinine, alongside reduced OI, were observed in non-survivors.

**Table 4:** Demographic and clinical characteristics of survivors and non-survivors in the severe COVID-19 group.

Variable	Survivors (*n* = 32)	Non-survivors (*n* = 34)	*p*-value
Age			
	67.50 (69.50–82.00)	78.00 (74.00–83.25)	0.28
Sex			
Female	9 (28.13%)	4 (11.76%)	0.28
Male	23 (71.88%)	30 (88.24%)	
BMI			
	24.05 (22.04–26.08)	23.44 (21.06–25.99)	0.70
Disease history			
Hypertension	19 (59.38%)	20 (58.82%)	0.99
Diabetes	8 (25.00%)	12 (35.29%)	0.66
CHD	3 (9.38%)	5 (14.71%)	0.83
Smoke history			
	3 (9.38%)	3 (8.82%)	0.99
Tumor biomarkers			
ProGRP (pg/mL)	58.50 (38.75–93.00)	69.50 (44.00–105.50)	0.26
CYFRA 21-1 (ng/mL)	6.48 (4.00–12.40)	12.15 (9.40–24.83)	8.8 × 10^−4^*
NSE (ng/mL)	24.50 (16.45–29.50)	26.60 (19.08–43.43)	0.14
CEA (ng/mL)	6.73 (3.31–11.99)	5.13 (3.88–11.46)	0.80
SCCA (ng/mL)	2.36 (1.30–4.11)	2.87 (1.40–3.97)	0.59
Laboratory test			
Lymphocyte ( × 10^9^/L)	0.45 (0.33–0.68)	0.50 (0.38–0.75)	0.30
Platelets ( × 10^9^/L)	171.00 (123.25–219.50)	183.50 (121.50–230.00)	0.16
CRP (mg/L)	97.96 (66.30–178.02)	149.20 (85.42–177.80)	0.16
ESR (mm)	45.50 (38.50–65.50)	51.00 (35.50–74.25)	0.72
ALT (U/L)	24.00 (16.00–51.00)	32.00 (23.30–40.13)	0.23
AST (U/L)	31.00 (20.50–49.25)	39.50 (30.90–68.40)	0.01*
proBNP (pg/mL)	603.90 (200.55–940.95)	1152.50 (606.93–2754.70)	0.003*
BUN (mmol/L)	6.45 (4.90–9.17)	8.85 (6.74–10.87)	0.02*
Cr (μmol/L)	62.45 (50.00–90.35)	92.00 (57.08–165.80)	0.03*
OI	197.50 (175.90–269.57)	177.73 (114.28–216.60)	0.02*

CHD, chronic heart diseases; ProGRP, progastrin-releasing peptide (ProGRP); CYFRA 21-1, cytokeratin 19 fragment; NSE, neuron-specific enolase (NSE); CEA, carcinoembryonic antigen; SCCA, squamous cell carcinoma antigen; CRP, C-reactive protein; ESR, erythrocyte sedimentation rate; ALT, alanine aminotransferase; AST, aspartate aminotransferase; proBNP, B-type natriuretic peptide precursor; BUN, blod urea nitrogen; Cr, creatinine; and OI, Oxygenation index.

Data are shown as median (IQR) or *n* (%). *p-value < 0.05 with statistical significance.

**Figure 3:**
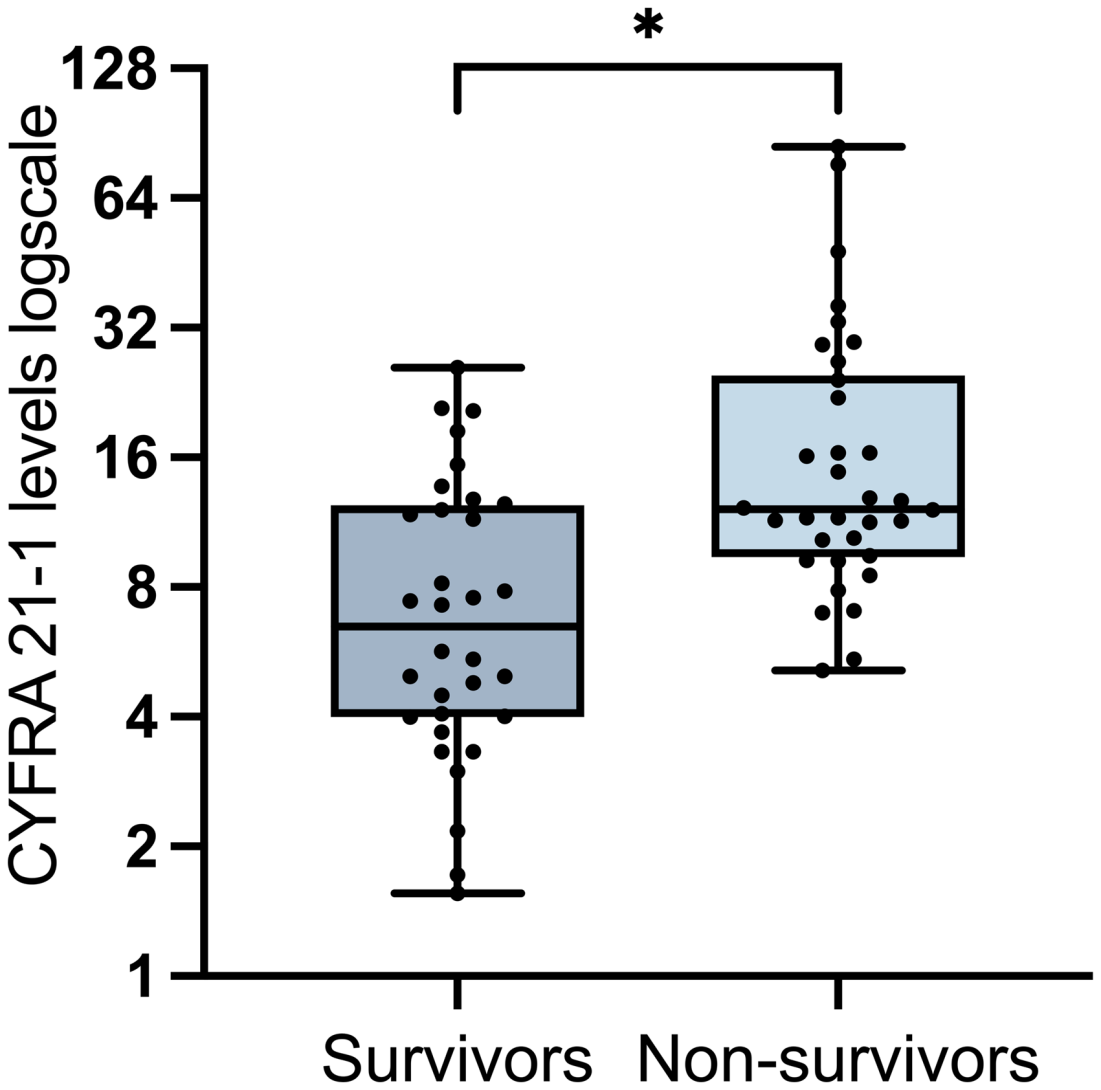
CYFRA 21-1 levels in survivors and non-survivors in the severe COVID-19 group. The survivors group consisted of patients discharged with improved condition, while the non-survivors group referred to those who experienced in-hospital mortality. Data are shown as median and range, with individual values indicated. *p-value < 0.05 with statistical significance.

**Figure 4:**
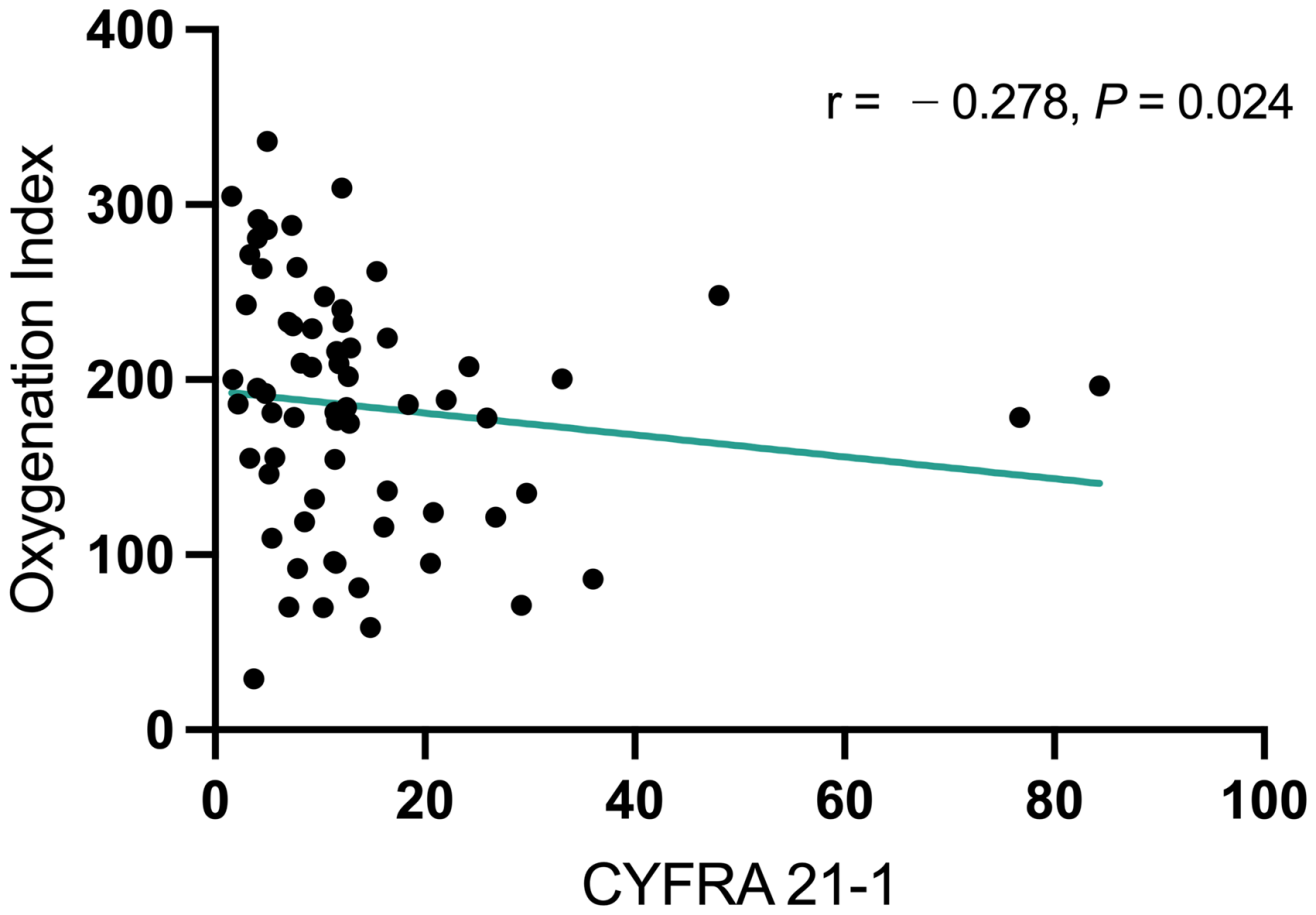
Spearman correlation analysis showing a negative correlation between CYFRA 21-1 levels and oxygenation index.

As illustrated in Figure 3, CYFRA 21-1 levels were notably elevated in the non-survivors group (*p* < 0.001), while other lung cancer–related biomarkers did not show considerable alterations (all *p >* 0.05). To determine whether CYFRA 21-1 could serve as an independent predictor of mortality, a multivariate logistic regression analysis was performed. CYFRA 21-1 predicted mortality with an OR value of 1.109 and a 95% CI of 1.036–1.225 (*p* = 0.02) (Table 5). Given that OI is a direct indicator of the severity of pneumonia, Spearman correlation analysis was performed to examine the relationship between CYFRA 21-1 levels and OI. CYFRA 21-1 levels were negatively correlated with OI, with a correlation coefficient of −0.278 (*p* = 0.024) (Figure 4). This finding suggests that elevated CYFRA 21-1 probably reflected greater alveolar epithelial damage, compatible with worse oxygenation.

**Table 5:** Multivariate logistic regression analysis of risk factors for mortality.

Variable	OR (Odds Ratio)	95% CI (Confidence Interval)	*p-*value
Tumor biomarkers			
CYFRA 21-1	1.109	1.036–1.225	0.02*
Laboratory test			
proBNP	1.001	1.000–1.001	0.08
Cr	1.014	1.002–1.030	0.047*

CYFRA 21-1, cytokeratin 19 fragment; proBNP, B-type natriuretic peptide precursor; BUN, urea nitrogen; and Cr, creatinine.

*p-value < 0.05 with statistical significance.

## Discussion

Unlike typical CAP, COVID-19 pneumonia can progress rapidly, making timely diagnosis crucial. Although diagnosis might seem straightforward, it can be complicated by unclear exposure histories or false-negative results in RT-PCR testing. Conversely, during the SARS-CoV-2 outbreak, many cases of bacterial pneumonia were also misdiagnosed as COVID-19 (7, 21). Early differentiation and clear risk stratification are essential, given that delayed treatment may lead to adverse outcomes. In this retrospective study, we compared COVID-19 pneumonia with CAP and found that CYFRA 21-1 exhibited notable diagnostic and prognostic value, reflecting its potential role in clinical management of COVID-19 pneumonia.

CYFRA 21-1 is a circulating fragment of cytokeratin 19. It is released during airway epithelial injury, offering a valuable biomarker reflecting epithelial cell disruption and turnover (18). Although CYFRA 21-1 was initially identified as a prognostic biomarker of non-small cell lung cancer, recent studies have demonstrated that elevated serum CYFRA 21-1 is associated with pulmonary complications in polytrauma patients, revealing its role as an early biomarker of acute lung injury (22, 23). Consistent with this, substantially increased levels of lung cancer-related biomarkers, including ProGRP, NSE, CYFRA 21-1, CEA, and SCCA, have also been reported (12, 24). Extending these findings, our data demonstrated the exceptional discriminative ability of CYFRA 21-1 among patients with non-COVID-19 CAP, mild COVID-19, survivors of severe COVID-19, and non-survivors of severe COVID-19. Moreover, within the cohort of patients with severe COVID-19 pneumonia, CYFRA 21-1 levels were negatively correlated with OI. This is consistent with earlier findings indicating that CYFRA 21-1 levels may reflect the extent of pulmonary epithelial damage (15, 18).

In this study, patients in the severe COVID-19 pneumonia group were significantly older, a factor known to influence the formation and maturation of immune cells (25). This may partially explain the pronounced lymphopenia observed in severe cases. In addition, older age has been linked to increased serum levels of cancer biomarkers (26, 27). Therefore, age-related differences must also be considered when interpreting these results. Although we adjusted for age in multivariate models and we detected no significant multicollinearity, residual confounding cannot be entirely excluded. Similarly, the severe group contained a higher proportion of male patients, consistent with previous studies, which may have also contributed to the variation in biomarker expression (24).

This study has several limitations. First, this was a single-center study with a relatively small sample size, which may limit its generalizability. Second, although the CAP cases were clinically assessed as bacterial pneumonia by at least three senior physicians, the presence of undetected viral infections cannot be completely excluded. Such unrecognized viral pneumonia could have limited diagnostic specificity when comparing COVID-19 and CAP. Furthermore, given that severe viral pneumonias such as influenza, Middle East respiratory syndrome, and respiratory syncytial virus infection exhibit comparable patterns of diffuse alveolar epithelial injury, it is plausible that CYFRA 21-1 could be similarly elevated in other types of severe viral pneumonia (27–30). Third, many patients with predisposing diseases were excluded, resulting in no inclusion of patients with severe CAP pneumonia in this study. Lastly, CYFRA 21-1 has been reported to be elevated in patients with interstitial lung disease, and since post-COVID-19 pulmonary fibrosis is common, it is intriguing whether CYFRA 21-1 is related to this condition. However, we only recorded data at the time of patient admission and did not conduct follow-up with survivors; therefore, this question remains unaddressed. Despite these limitations, to our knowledge, this study is the first to demonstrate that CYFRA 21-1 not only distinguishes COVID-19 pneumonia from regular CAP but also serves as an independent prognostic predictor for COVID-19 outcomes. Hopefully, testing for CYFRA 21-1 levels can be a practical tool to assist front-line doctors in better diagnosing and managing COVID-19 patients.

## Conclusion

Our data suggest a potential role for CYFRA 21-1 as both a diagnostic and prognostic biomarker in COVID-19 pneumonia. Further research with larger, multicenter, and different viral infection cohorts, including follow-up data, is warranted to validate these findings and explore the association of CYFRA 21-1 with post–COVID-19 pulmonary conditions.

## Data Availability

The raw data supporting the conclusions of this article will be made available by the authors, without undue reservation.
