# Supplementary material for: Clinical significance of cytokeratin 19 fragment in COVID-19 patients: a retrospective study
Source: Front Public Health. 2025 Dec 18;13:1738947. doi: 10.3389/fpubh.2025.1738947 (PMC12756477; doi:10.3389/fpubh.2025.1738947)
Supplement: Supplementary file 1 [file Supplementary_file_1.docx]

**Supplementary Figure S1. CONSORT Flow Diagram.**

Patients admitted for community-acquired pneumonia between December 2022 to September 2023

(n = 707)

390 patients excluded with a

history of tumors or structural lung diseases

317 patients were divided into two groups: COVID-19 and non-COVID-19

20 patients excluded with length of stay < 3 days

20 patients excluded with lacking laboratory tests

24 patients excluded with the final diagnosis of organized pneumonia

Non-COVID-19

(n = 87)

COVID-19

(n = 166)

1 patient excluded with

critical psittacosis

Mild non-COVID-19 CAP

(n = 86)

Severe COVID-19 pneumonia

(n = 66)

Mild COVID-19 pneumonia (n = 100)

**Table S1. Univariate analysis of clinical variables between mild CAP and mild COVID-19 pneumonia.**

|  | **OR**  **(odds ratio)** | **95% CI**  **(confidence interval)** | ***P* - value** |
| --- | --- | --- | --- |
| **Tumor biomarkers** | | | |
| *CYFRA 21-1* | 2.556 | 1.821–3.872 | ＜ 0.001* |
| *CEA* | 1.289 | 1.093–0.511 | ＜ 0.001* |
| **Laboratory test** | | | |
| *Lymphocyte* | 0.301 | 0.168–0.079 | ＜ 0.001* |
| *Platelets* | 0.990 | 0.985–0.994 | ＜ 0.001* |

CYFRA 21-1, cytokeratin 19 fragment and CEA, carcinoembryonic antigen. * *P*-value < 0.05 with statistical significance.

**Table S2. Univariate logistic regression analysis for risk factors associated with the severity of COVID-19 pneumonia.**

|  | **OR**  **(Odds Ratio)** | **95% CI**  **(Confidence Interval)** | ***P* - value** |
| --- | --- | --- | --- |
| **Gender** | | | |
|  | 4.786 | 2.377–10.174 | ＜ 0.001* |
| **Age** | | | |
|  | 1.070 | 1.039–1.106 | ＜ 0.001* |
| **Tumor biomarkers** | | | |
| *ProGRP* | 1.027 | 1.016–1.040 | ＜ 0.001* |
| *CYFRA 21-1* | 1.677 | 1.443–1.982 | ＜ 0.001* |
| *NSE* | 1.170 | 1.111–1.244 | ＜ 0.001* |
| *CEA* | 1.228 | 1.126–1.359 | ＜ 0.001* |
| *SCCA* | 1.180 | 1.060–1.384 | 0.016* |
| **Laboratory test** | | | |
| *Lymphocyte* | 0.026 | 0.007–0.079 | ＜ 0.001* |
| *CRP* | 1.023 | 1.016–1.031 | ＜ 0.001* |
| *ESR* | 1.025 | 1.012–1.039 | ＜ 0.001* |
| *ALT* | 1.008 | 0.998–1.017 | 0.115 |
| *AST* | 1.011 | 1.001–1.019 | 0.059 |
| *proBNP* | 1.002 | 1.001–1.003 | ＜ 0.001* |
| *BUN* | 1.371 | 1.211–1.584 | ＜ 0.001* |

ProGRP, progastrin-releasing peptide; CYFRA 21-1, cytokeratin 19 fragment; NSE, neuron-specific enolase; CEA, carcinoembryonic antigen; SCCA, squamous cell carcinoma antigen; CRP, C-reactive protein; ESR, erythrocyte sedimentation rate; ALT, alanine aminotransferase; AST, aspartate aminotransferase; proBNP, B-type natriuretic peptide precursor; and BUN, blood urea nitrogen. * *P*-value < 0.05 with statistical significance.

**Table S3. Univariate analysis of clinical variables between survivors and non-survivors among patients with severe COVID-19 pneumonia.**

|  | **OR (Odds Ratio)** | **95% CI (Confidence Interval)** | ***P* - value** |
| --- | --- | --- | --- |
| **Tumor biomarkers** | | | |
| *CYFRA 21-1* | 1.138 | 1.054–1.258 | 0.007* |
| **Laboratory test** | | | |
| *AST* | 1.009 | 1.000–1.027 | 0.171 |
| *proBNP* | 1.001 | 1.000–1.001 | 0.017* |
| *BUN* | 1.091 | 0.999–1.228 | 0.091 |
| *Cr* | 1.015 | 1.005–1.029 | 0.013* |

CYFRA 21-1, cytokeratin 19 fragment; AST, aspartate aminotransferase; proBNP, B-type natriuretic peptide precursor; BUN, blood urea nitrogen; and Cr, creatinine.

* *P*-value < 0.05 with statistical significance.
